# GC/TOF-MS-Based Metabolomics Reveals Altered Metabolic Profiles in Wood-Feeding Termite *Coptotermes formosanus* Shiraki Digesting the Weed *Mikania micrantha* Kunth

**DOI:** 10.3390/insects12100927

**Published:** 2021-10-11

**Authors:** Wenjing Wu, Yahui Hou, Shijun Zhang, Yong Chen, Wenhui Zeng, Zhiqiang Li

**Affiliations:** 1 Guangdong Key Laboratory of Animal Conservation and Resource Utilization, Guangdong Public Laboratory of Wild Animal Conservation and Utilization, Institute of Zoology, Guangdong Academy of Sciences, Guangzhou 510260, China; wuwj@giabr.gd.cn (W.W.); zhangsj@giabr.gd.cn (S.Z.); stout_chen@giabr.gd.cn (Y.C.); zengwh@giabr.gd.cn (W.Z.); 2 Guangzhou Institute of Forestry and Landscape Architecture, Guangzhou 510405, China; houyahui0920@163.com

**Keywords:** *Coptotermes formosanus*, *Mikania micrantha*, gas chromatography/time-of-flight mass spectrometry (GC/TOF-MS), metabolic profiles, dietary intake, mortality

## Abstract

**Simple Summary:**

*Mikania micrantha* Kunth is among the most invasive weeds in the world, causing extensive damage to both natural ecosystems and agroforestry systems. Mechanical removal is one of the most effective and straightforward approaches to controlling this weed, but this results in extensive lignocellulosic waste, and effective approaches to exploiting this abundant biomass are limited. *Coptotermes formosanus* Shiraki is not only an important subterranean termite pest species but also a considerable decomposer with the ability to digest lignocellulose. In this study, we evaluated the effects of a diet composed of *M. micrantha* leaves on *C. formosanus* workers. The workers increased their dietary intake when fed *M. micrantha* leaves, with a concomitant gradual increase in mortality rate. From the metabolic profiles, changes in metabolites and their related metabolic pathways suggested that termites can utilize *M. micrantha*-derived lignocellulose, but their antioxidant activity and signal transduction may be suppressed. Overall, this study identified key metabolites and pathways associated with the response of these termites to dietary changes and the effect of *M. micrantha* on termites.

**Abstract:**

Effective approaches to exploiting the biomass of the abundant invasive weed *Mikania micrantha* Kunth are limited. Termites have been a focus of significant attention as mediators of biomass-processing owing to their ability to digest lignocellulose. Here, the GC/TOF-MS approach was employed to assess the effects of a diet composed of *M. micrantha* leaves on *Coptotermes formosanus* workers, with the growth performance of these workers also being assessed. The workers increased their dietary intake when fed *M. micrantha* leaves, with a concomitant gradual increase in mortality rate. A total of 62 differentially abundant metabolites and nine significantly affected pathways were found when comparing termites fed *M. micrantha* leaves to pinewood. Key metabolites, including carbohydrates, polyols, 4-hydroxyphenylacetic acid, and their related metabolic pathways, suggested that termites can digest and utilize *M. micrantha*-derived lignocellulose. However, changes in the tryptophan metabolism, tyrosine metabolism, and sphingolipid metabolism suggest an adverse effect of *M. micrantha* leaves on antioxidant activity and signal transduction in termites. Overall, this study identified the key metabolites and pathways associated with the response of these termites to dietary changes and the effect of *M. micrantha* on termites.

## 1. Introduction

Owing to their ability to decompose lignocellulose, termites are among the most ecologically important invertebrate detritivore species in the world [1]. Termites can feed on diverse substrates including wood, leaf litter, and highly humified soil residues [2]. Termites are commonly studied as a model organism by researchers evaluating the ability of species to adapt to specific forms of plant biomass degradation and to study the mechanisms of lignocellulose degradation. Prior studies have evaluated the physiological changes in termite survival, body mass, and symbiotic protist associations that occur when they are fed diets consisting of softwood, hardwood, or rice straw [3,4]. Termite host-symbiont meta-transcriptomic sequencing studies have revealed that specific differentially expressed ligninase, phenoloxidase, detoxification, and antioxidant transcripts are associated with the ability of these termites to process a diet rich in lignin [5]. Enzymatic activity in the guts of termites and associated symbiotic enzymatic activity, including endoglucanase, exoglucanase, xylosidase and glutathione peroxidase, has been shown to change in response to different diets composed of pinewood, corn stover, or soybean residue [6,7]. These dietary changes are also associated with shifts in the community richness and diversity of protists and bacteria within the gut microbiome of these termites, resulting in altered functional roles that facilitate optimal resource utilization [8,9,10,11,12,13]. These prior reports affirm the ability of termites and associated symbiotes to control physiological responses, gene expression, enzymatic activity, and community structural composition such that they are better able to adapt to a range of lignocellulosic diets. The biochemical metabolites associated with these dietary and regulatory shifts, in contrast, have not been well-characterized to date.

Metabolomics studies evaluate changes in small-molecule (<1500 Da) metabolites in a given biological system [14], with the changes in these metabolite levels directly reflecting shifts at the genetic or environmental levels, including changes in diet or lifestyle [14]. Metabolomics analyses of termite populations have been used to assess termite secretions, including soldier defensive secretions [15,16] and labial gland secretions from soldier and worker termites [17], as well as to monitor metabolic processes such as lignin digestion/modification [5,18,19,20,21] and cellulose catabolism [22]. Such metabolomics analyses thus offer a powerful approach to exploring the impact of different diets on metabolite levels in termites and can offer insight into the adaptation process.

*Mikania micrantha* Kunth (Asteraceae) is an invasive weed that is widely distributed throughout Southern China, Southeast Asia, and the Pacific Islands, where it has caused serious damage to forests and agricultural lands [23]. Mechanical removal is one of the most effective and straightforward approaches to eliminating *M. micrantha* infestations, but this results in extensive lignocellulosic waste [23]. The lignin and holocellulose content of the entirety of *M. micrantha* plants, not including the roots, are 19.1 ± 0.7 and 61.5 ± 1.4 wt% raw biomass, respectively [24]. The crude fiber from the leaves of these plants consists of 21.31 ± 1.56% cellulose, 14.61 ± 1.46% hemicellulose, 20.67 ± 0.8% lignin, 9.11 ± 0.23% ash, and 34.30 ± 1.15% impurities and moisture [25]. Moreover, aqueous extracts of *M. micrantha* leaves contain amino acids, proteins, saponins, flavonoids, and phenolic compounds [26,27]. Some phenolic and flavonoid compounds have been reported to exhibit antioxidant, antimicrobial, and anti-inflammatory properties [28]. As such, *M. micrantha* holds great promise as a sustainable food source for lignocellulolytic organisms and may also exert biological effects on these organisms, although the current understanding of these effects remains limited.

As one of the most evolved transitional taxa between the lower and higher termites [29], *Coptotermes formosanus* Shiraki species can feed on a range of lignocellulosic substrates and can be readily maintained under laboratory conditions [3,4,21,30]. Herein, we assessed changes in survival, dietary mass, and metabolite profiles in *C. formosanus* workers in response to diets composed of *M. micrantha* leaves or pinewood. This work was undertaken to understand the ability of wood-feeding termites to consume weed-based waste biomass and the beneficial and/or harmful effects of *M. micrantha* on termites, and offer additional insight into the development and utilization of *M. micrantha*-derived lignocellulose.

## 2. Materials and Methods

### 2.1. Termite Colony and Dietary Preparation

Two *C. formosanus* colonies collected from Guangzhou International Biotech Island and South China University of Technology campus (Guangzhou, Guangdong Province, China) were used for this study. No specific permissions were required to utilize colonies from these locations. Soldier or alate morphology was used to facilitate species identification. Worker termites used for this study were identified based upon the absence of wing buds and abdominal distension. Individual colonies were placed into separate plastic containers containing a diet of pinewood (*Pinus massoniana* Lamb., a mixture of sapwood and heartwood obtained from Sheng Wenjie Wood Products, Guangzhou, Guangdong Province, China), and were maintained for at least one month in a climate-controlled chamber (Ningbo Jiangnan Instrument Factory, Ningbo, China) (28 ± 1 °C, 80 ± 5% relative humidity).

To prepare an experimental *M. micrantha* leaf (ML) diet, leaves of *M. micrantha* were collected from Sun Yat-sen University campus in Guangzhou, Guangdong Province, China. These leaves were separated, allowed to air dry in an open area for one week, and ground to a fine powder. This powder was then compressed to yield 3.5 cm diameter ML “cookies”. Cookies were dried for 48 h at 50 °C and weighed prior to feeding. A pinewood (PW) diet was prepared using these same methods as an experimental control.

### 2.2. Bioassays

Three replicates of each diet were tested for each of the two studied termite colonies to yield six replicates. The selected worker termites were sound and similar in size. Each diet was placed in the center of a 50 mL glass beaker and saturated with deionized water. A total of 150 termite workers and 15 soldiers were added to each beaker after having been starved for 24 h. Beakers were incubated in a climate-controlled chamber (28 ± 1 °C and 80 ± 5% relative humidity), with deionized water being added to the diet on days 2, 4, 6, and 8 to prevent desiccation. The number of live workers was measured once per day for 10 days. At the end of this 10-day period, any remaining food was collected, dried in an oven, and weighed to quantify diet consumption. The effects of termite colony on bioassay results were assessed, revealing no significant differences between similar colonies (Table S1). Student’s *t*-tests was used to compare differences in termite survival or dietary intake between groups, and the generalized linear model (quasi-Binomial distribution) was used to analyze the effects of diet and time on cumulative termite mortality, using the SPSS Statistics 22 software (IBM, Armonk, NY, USA) (*p*-values < 0.05 as the significance threshold).

### 2.3. Metabolite Sample Collection and Preparation

For GC/TOF-MS (gas chromatography/time-of-flight mass spectrometry) analyses, 130 *C. formosanus* workers and 13 soldiers were fed ML and PW diets as above, with six replicates per treatment. At the end of the 10-day treatment period, 100 workers were selected at random from each beaker, snap-frozen with liquid nitrogen, and stored at −80 °C. A 30 mg aliquot of each sample was then ground into a fine powder in liquid nitrogen, after which chloroform and methanol were used for extraction. l-2-Chlorophenylalanine was added to each sample as an internal standard. Samples were homogenized at 45 Hz for 4 min in a ball mill, ultrasonicated for 5 min in an ice water bath, and subjected to centrifugation for 15 min at 13,000× *g* at 4 °C. Supernatants were then transferred into fresh GC/MS glass vials and dried in an unheated vacuum concentrator. Each dried extract was then combined with 30 μL of methoxyamination hydrochloride (20 mg/mL in pyridine), followed by incubation at 80 °C for 30 min. Samples were then derivatized for 90 min using bistrimethyl-silyl-triflouroacetamide (BSTFA) containing 1% trimethylchlorosilane (TMCS) (40 μL) at 70 °C.

### 2.4. GC/TOF-MS Analysis

An Agilent 7890B gas chromatograph instrument (Agilent Technologies, Santa Clara, CA, USA) equipped with a Pegasus HT time-of-flight mass spectrometer (LECO Corporation, St Joseph, MI, USA) was used to conduct GC/TOF-MS analyses with a DB-5MS capillary column coated with 5% diphenyl cross-linked with 95% dimethylpolysiloxane (30 m × 250 μm inner diameter, 0.25 μm film thickness; J&W Scientific, Folsom, CA, USA). A 1 μL sample volume was injected in splitless mode, with helium as a carrier gas at a flow rate of 1 mL/min. The column was maintained at 50 °C for 1 min, after which it was heated at 10 °C min^−1^ to 310 °C and then maintained at this temperature for 8 min. The temperatures of the injection, transfer line, and ion source were 280 °C, 270 °C, and 220 °C, respectively. The energy was −70 eV in electron impact mode. MS data were acquired in full-scan mode at 20 spectra per second following a 455 s solvent delay with a mass-to-charge ratio (*m/z*) range of 50–500.

### 2.5. Metabolomic Data Analysis

Raw GC/TOF-MS data were analyzed as in prior reports [31]. Raw peaks were extracted, filtered, calibrated, aligned, identified, and peak areas were integrated using the Chroma TOF 4.3X software (LECO Corporation) and the LECO-Fiehn Rtx5 database [31]. Metabolites that were detected in <50% of QC samples were excluded from further analysis [32]. Results were normalized using internal standards. Similarity values (SVs) were used to assess the accuracy of a given compound, with SV values greater than 700 indicating high-confidence metabolite identification, while an SV < 200 was considered to indicate that a metabolite identification was “unreliable”, and an SV between 200 and 700 was considered a tentative annotation.

The resultant 3D data, including peak number, sample name, and normalized peak area, were introduced into the SIMCA 14.1 software package (V14.1, MKS Data Analytics Solutions, Umea, Sweden) to facilitate principal component analysis (PCA), partial least squares-discriminant analysis (PLS-DA), and orthogonal projections to latent structures-discriminate analysis (OPLS-DA). Candidate metabolites of interest were selected based upon Variable Importance for the Projection (VIP) values extracted from the first principal component in the OPLS-DA analysis. Significantly differentially abundant metabolites, which were those that were most reliably able to differentiate termite treatment groups, were identified based on VIP value > 1.0 and a *p*-values < 0.05 (Student’s *t*-test). KEGG pathways associated with these metabolites were identified using MetaboAnalyst 5.0 (http://www.metaboanalyst.ca, accessed on 14 August 2021) [33]. Significantly enriched KEGG pathways were elected based on *p*-values from pathway enrichment analyses or impact values for pathway topological analyses [34,35]. Impact values > 0.1 and negative log(*p*) values > 2.0 served as significance threshold for this analysis.

## 3. Results

### 3.1. ML Diets Increase Food Intake While Decreasing Survival Rates

**Figure 1:**
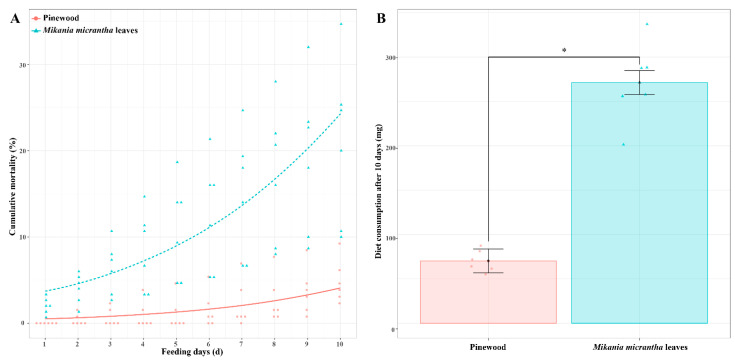
Termite cumulative mortality (**A**) and dry weight consumption of diets (**B**) after *C. formosanus* workers were fed a *M. micrantha* leaves or pinewood diet for 10 days. Red and blue dots represent the original data of each replicate from termites fed a pinewood diet and a *M. micrantha* leaves diet, respectively (*n* = 6). Error bars represent standard deviations; asterisk indicates *p* < 0.05.

We began by assessing termite survival and dietary mass consumed for *C. formosanus* workers fed a *M. micrantha* leaves (ML) or pinewood (PW) diet. Survival rates for termites fed an ML diet for 10 days (79.11 ± 0.09%) were significantly lower than those for termites fed a PW diet (95.13 ± 0.02%) (*t* = 4.01, *p* < 0.01, *n* = 6). Termite cumulative mortality was significantly impacted by dietary composition (*χ*^2^ = 208.79, *p* < 0.001, *n* = 6) and the number of days (*χ*^2^ = 117.16, *p* < 0.01, *n* = 6), but was not affected by their interaction (*χ*^2^ = 2.29, *p* = 0.13, *n* = 6) (Figure 1A). Moreover, *C. formosanus* workers consumed 271.3 mg of the ML diet, with this amount being 3.86-fold higher than the amount of biomass consumed by termites fed a PW diet (70.2 mg) (*t* = 10.52, *p* < 0.001, *n* = 6) (Figure 1B).

### 3.2. GC/TOF-MS Analyses Reveal Diet-Specific Metabolic Profiles

We hypothesized that changes in *C. formosanus* physiology associated with the differential feeding performance observed above would be reflected by metabolite levels in these animals. GC/TOF-MS analyses yielded 766 valid peaks, of which 731 were retained following quality control filtering. Using the LECO-Fiehn Rtx5 Metabolomics Library, 299 metabolites were quantified in these samples, including 66 with an SV > 700 and 227 metabolites with an SV of 200–700.

**Figure 2:**
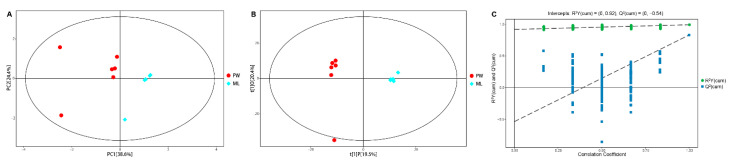
PCA score plot (**A**) and OPLS-DA score plot (**B**) with corresponding permutation test plot (**C**) derived from the GC/TOF-MS metabolite profiles of *C. formosanus* workers. Red and blue dots indicate metabolite profile of termites fed a pinewood (PW) diet and a *M. micrantha* leaves (ML) diet, respectively; a dot represents one replicate (*n* = 6); the circle represents the 95% Hotelling’s T-squared ellipse.

To decrease the overall complexity of the resultant metabolite dataset, multivariate PCA and OPLS-DA analyses were conducted, enabling the visualization of similarities and differences between treatment groups. PCA analyses of these GC/TOF-MS metabolic profiles revealed clear separation between termites fed ML and PW diets, with PC1 and PC2, respectively accounting for 38.6% and 24.4% of the overall variation (Figure 2A). Relative to the PW diet, the R^2^X value of the PCA model representing the explained variance was 0.794. All samples in the resultant score plots fell within the 95% Hotelling’s T-squared ellipse, consistent with an absence of any outliers among these evaluated samples.

To achieve maximal discrimination between the ML and PW groups, OPLS-DA was used to better clarify metabolic patterns in these termites (Figure 2B). Parameters considered for classification from the software were R^2^X = 0.399, R^2^Y = 0.995, and Q^2^ = 0.833, all of which were stable and effective for fitness and prediction. Respective R^2^ and Q^2^ intercept values determined following 200 permutations were 0.92 and −0.54 (Figure 2C). The low Q^2^ intercept value is indicative of the robustness and reliability of these models and suggests that the risk of overfitting is low. Both of these multivariate analyses revealed clear differences in metabolite profiles from termites fed ML and PW diets, confirming that there are significant metabolic differences between these diets.

### 3.3. Metabolite Identification and Comparison

**Figure 3:**
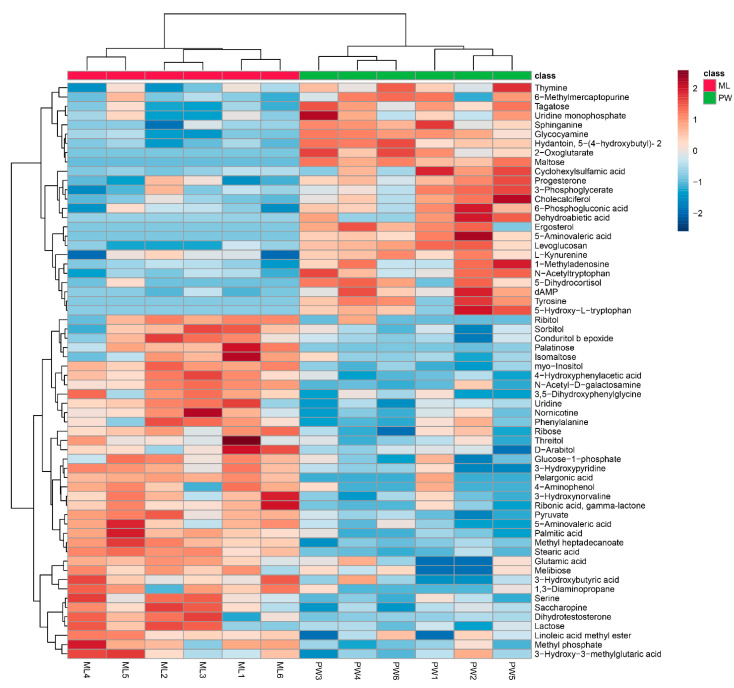
Hierarchical clustering analysis for significantly differentially abundant metabolites. The relative metabolite level is depicted according to the color scale. Red and blue indicate upregulated and downregulated metabolites in termites fed a *M. micrantha* leaves (ML) diet relative to those fed a pinewood (PW) diet, respectively.

Changes in metabolite levels in termites fed an ML diet were compared to those in termites fed a PW diet based upon fold-change (FC) values. Based upon VIP threshold cut-offs (VIP > 1.0; *p* < 0.05), a total of 62 metabolites were found to be differentially abundant between these two termite groups (Figure 3), of which 37 were upregulated in termites fed an ML diet relative to those fed a PW diet (Table 1). These metabolites included carbohydrates (e.g., palatinose, isomaltose, lactose, melibiose), polyols (e.g., *myo*-inositol, d-arabitol, sorbitol), amino acids and derivatives thereof (e.g., serine, glutamic acid, 4-hydroxyphenylacetic acid), and fatty acids (e.g., pelargonic acid, palmitic acid) (Table 1). Nevertheless, some significantly increased metabolites in Table 1 were downregulated in a few ML group replicates, e.g., serine in ML6, methyl phosphate in ML3, isomaltose in ML4/5, d-arabitol in ML2, sorbitol in ML4, phenylalanine in ML5, and ribose in ML3 (Figure 3). An additional 25 metabolites were significantly downregulated in termites fed an ML diet, including tyrosine, 2-oxoglutarate, 5-aminovaleric acid, 5-hydroxytryptophan, kynurenine, maltose, and tagatose (Table 2). Likewise, changes in some metabolites in each replicate were not entirely consistent, as for thymine, 6-methylmercaptopurine, tagatose, uridine monophosphate, 6-phosphogluconic acid, and 5-dihydrocortisol in ML5 (Figure 3).

### 3.4. Metabolic Pathway Analyses of Differentially Abundant Metabolites

**Figure 4:**
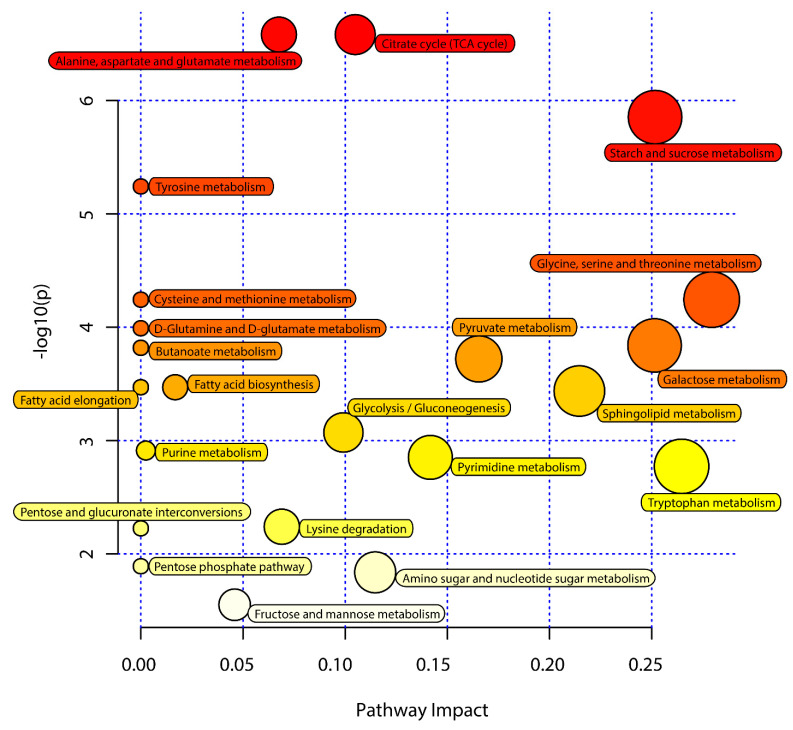
Pathway analysis of the *M. micrantha* leaves (ML) diet and pinewood (PW) diet groups. The *y*-axis represents negative log(*p*) values from pathway enrichment analysis, the *x*-axis represents impact values from the pathway topology analysis. The color and size of the shapes represent the effects of the ML diet on termite metabolism relative to the PW diet; large, red shapes indicate a greater effect on the pathway.

**Figure 5:**
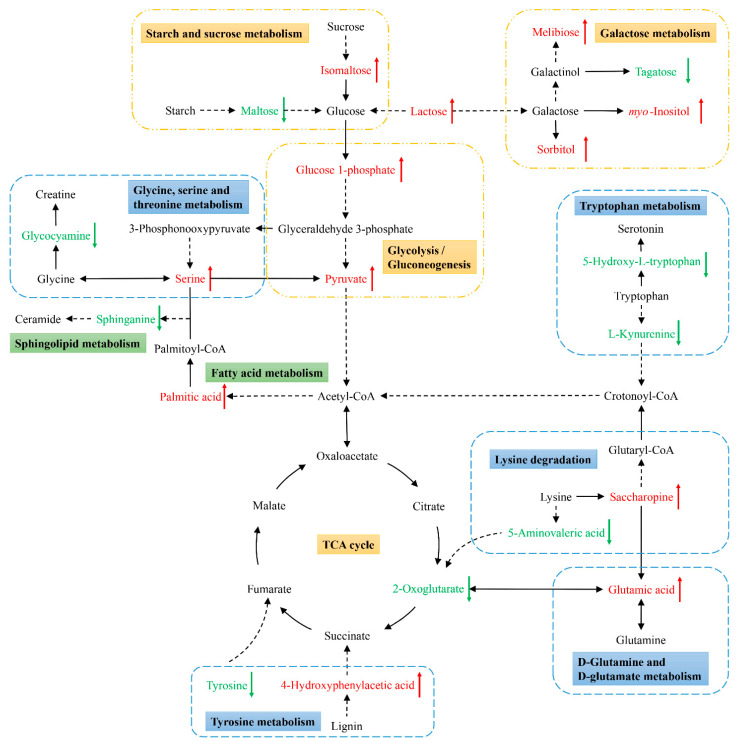
Schematic overview of the primarily affected metabolic pathways in *C. formosanus* workers. The red characters indicate metabolites that were highly abundant in termites fed a *M. micrantha* leaves diet, while green ones indicate metabolites that were highly abundant in termites fed a pinewood diet. Solid and dotted line arrows indicate direct and indirect reactions, respectively.

Next, KEGG pathway analyses of the differentially abundant metabolites identified above were performed using MetaboAnalyst 5.0 to identify significantly enriched metabolic pathways associated with the consumption of an ML diet. In total, 33 of these metabolites were associated with 22 enriched metabolic pathways when analyzing samples from these *C. formosanus* workers (Figure 4, Table 3). Of these pathways, 9 exhibited pathway impact values exceeding 0.1, which was the designated relevance threshold, following the topological and enrichment analyses of these identified pathways (Table 3). The most enriched pathways associated with these differentially abundant metabolites, as determined based upon negative log(*p*) and pathway impact values, were the glycine, serine and threonine metabolism, tryptophan metabolism, starch and sucrose metabolism, and galactose metabolism pathways, all of which had impact values of greater than 0.25 (Figure 4). A schematic overview of these pathways was generated using a reference map derived from the KEGG database (Figure 5). These differentially enriched metabolic pathways were primarily associated with the metabolic processing of amino acids, carbohydrates, fatty acids, and nucleosides.

## 4. Discussion

In this study, a diet consisting of *M. micrantha* leaves was found to increase *C. formosanus* workers food intake but to decrease their survival rates. Following a switch to this food source, these termites exhibited adjustments in their nutrient requirements, metabolic activity, stress responses, and signal transduction at the metabolic level. These results can advance current understanding regarding metabolic shifts associated with dietary alteration in termites and potential utilization of *M. micrantha*-derived lignocellulose.

Previous grass-diet (e.g., rice straw, corn stover, and soybean residue) studies in lower termites, *C. formosanus* and *Reticulitermes flavipes*, have demonstrated a positive correlation between food intake and survival rate [13,21]. The survival rate of termites fed an ML diet in this study was slightly higher than previously reported rates associated with a rice straw diet and lower than rates associated with diets consisting of corn stover or soybean residue, although the dietary intake was higher than for these grass-based diets. Relative to pinewood, *M. micrantha* leaves contain less cellulose (ML: 21.31%, PW: 43.12–49.25%) and lignin (ML: 20.67%, PW: 26.12–35.42%) content [25,36], potentially leading to the increased ML dietary intake by these termites such that they were able to sustain themselves on this diet. However, this increase in overall dietary intake by termites fed an ML diet may have also resulted in the increased intake of toxic secondary plant substances [37], resulting in adverse survival outcomes.

Metabolic profiling and functional analyses of associated metabolic pathways provided insight regarding differences in metabolic status associated with the two experimental diets in the present study (Figure 5). Changes in certain metabolites in these analyses may be indicative of increased biosynthesis and utilization of related substances in these termites. For example, there were relatively high levels of glutamic acid, a key metabolite that links carbon and nitrogen metabolism [22], enabling further utilization in a range of biochemical processes. Worker termites fed an ML diet exhibited significant increases in the abundance of hydrolysates, glycolytic pathway metabolites, sugars, and alcohols. Some of these carbohydrates and their derivatives such as sorbitol, melibiose, and inositol can be further used by gut bacteria to synthesize acids [38]. The metabolite 4-hydroxyphenylacetic acid, a key intermediate associated with the lignin degradation, can be broken down into the TCA cycle intermediate succinate to yield energy via the homoprotocatechuate degradation pathway by termite symbionts [39,40]. Additionally, the carbon-to-nitrogen (C:N) ratio of *M. micrantha* (around 25:1) is much lower than of *p. massoniana* (around 673:1), which suggests that *M. micrantha* may contain much more protein than pinewood [41,42]. Prior to collection, starch had been synthesized in the *M. micrantha* leaves by photosynthesis with various enzymes (proteins). In light of these facts, the enrichment of the glycine, serine and threonine metabolism, tryptophan metabolism, and starch and sucrose metabolism pathways may also be attributable to the digestion of the proteins and starch present within *M. micrantha* leaves. Accordingly, the changes in these metabolites and pathways suggest that termites together with their symbionts increase their digestion and utilization of *M. micrantha*-derived lignocellulose and other substrates.

Changes in the levels of certain metabolites may be indicative of shifts in the physiology or behavioral status of the analyzed termites. For example, levels of two key metabolites associated with tryptophan metabolism, kynurenine and 5-hydroxy-l-tryptophan (5-HTP), were significantly reduced in termites fed an ML diet. Kynurenine can control responses to environmental stress, dietary changes, or infection [43,44,45,46]. Additionally, 5-HTP has been shown to be closely linked to important physiological processes affecting locomotor activity, mood, and feeding behavior [47,48,49]. Reductions in kynurenine and 5-HTP levels in termites fed an ML diet may indicate that these termites are in a stressed or nutrient-deprived state, prompting increased food intake. Lysine is degraded to glutaryl-CoA by the major saccharopine pathway, ultimately yielding acetyl-CoA and carbon dioxide [50]. Increased lysine degradation is evident in starved insects [51,52], further suggesting that termites fed an ML diet may be in a starvation state. A TCA cycle intermediate in the context of ATP or GTP production, 2-Oxoglutarate has been linked to important biological activities including lipid peroxidation and ammonia detoxification, in addition to serving as a precursor for amino acid and nucleotide synthesis [53,54]. Decreases in 2-oxoglutarate levels may be indicative of a shortage of intermediate compounds and energy in termites fed an ML diet. As such, metabolites related to the nutritional/energy status or feeding behaviors of termites may undergo adjustment in response to shifts in dietary composition.

Changes in the levels of certain metabolites may be characteristic of changes in antioxidant, immunological, signal transduction, and other metabolic processes in termites fed an ML diet. Tyrosine can serve as a neurotransmitter, antioxidant, and plays a key role in the context of protein modification [55,56,57]. Decreased tyrosine abundance may be associated with a consequent reduction in antioxidant activity and immune function in termites fed an ML diet. The reduction in 5-HTP levels associated with tryptophan metabolism may impact the biosynthesis of serotonin and melatonin [57,58], thus limiting their ability to suppress inflammation and superoxide activity following ML dietary intake. Ceramide is a bioactive sphingolipid that regulates key cellular processes such as proliferation, differentiation, and apoptosis [59]. Ceramide biosynthesis occurs via the initial condensation of serine and palmitic acid and the formation of sphinganine as an intermediate molecule [60]. Termites fed an ML diet exhibited increases in serine and palmitic acid abundance that did not translate to an increase in sphinganine levels in these animals, suggesting the disruption of this biosynthetic process. As such, these altered metabolite levels may be indicative of a decreased ability of these termites to respond to the harmful ingredients (such as phenolic and flavonoid compounds) in an ML diet, further impairing their survival.

Furthermore, termite symbionts not only participate in the digestion of lignocellulose, but may also be affected by the diet. Because lignin has positive effects on the maintenance of termite symbionts [3], the low lignin content of *M. micrantha* leaves may influence the structure and function of gut symbionts. For instance, the downregulation of 5-aminovaleric acid may be one of the indicators, which is an important intermediate during bacteria catabolize lysine via the 5-aminopentanoate (5-aminovaleric acid) pathway to obtain carbon, nitrogen, and energy [61].

## 5. Conclusions

Our results demonstrate the ability of *C. formosanus* workers to feed on *M. micrantha* leaves and to metabolize lignocellulose and other substrates derived from these leaves. According to their metabolic profiles, *M. micrantha* leaf-based diets may promote increased feeding behavior and food consumption in termites, but may also adversely suppress termite responses to harmful substances and have chronic toxicity. Hence, *C. formosanus* cannot be fed on *M. micrantha* for a long time, and it would be inappropriate for *M. micrantha* to be a single feed source of *C. formosanus*. It may be possible to utilize *M. micrantha* as an additive in termite baits. Further research, however, is necessary to more fully establish the roles of these metabolites in other dietary contexts and to determine how they relate to changes in gene expression.

## Figures and Tables

**Table 1:** Significantly differentially abundant metabolites that were increased in termites fed a *M. micrantha* leaves diet relative to those fed a pinewood diet.

Metabolites	Similarity	Mass	VIP	*p*-Value	Fold Change
*myo*-Inositol	847	318	2.0983	0.0000	2.6153
Serine	843	218	1.7328	0.0056	1.9249
Methyl phosphate	836	241	1.7052	0.0048	1.5119
Isomaltose	832	361	1.3743	0.0425	1.8170
d-Arabitol	823	217	1.3186	0.0317	1.5240
Sorbitol	822	307	1.3696	0.0281	1.5178
Phenylalanine	821	218	1.3800	0.0390	1.5033
Ribose	815	103	1.5596	0.0128	1.3338
3-Hydroxypyridine	810	152	1.2158	0.0011	2.9223
Palmitic acid	806	313	1.9743	0.0003	1.4083
Stearic acid	788	341	2.1285	0.0000	1.7277
3-Hydroxybutyric acid	754	147	1.1377	0.0208	2.4876
Glutamic acid	744	246	1.0792	0.0327	1.9142
Threitol	704	217	1.4558	0.0424	1.6505
Ribitol	670	217	1.5749	0.0147	5.4272
Pyruvate	670	174	1.7218	0.0002	2.7589
*N*-Acetyl-d-galactosamine	667	202	1.9062	0.0006	2.8560
Glucose-1-phosphate	660	217	1.6018	0.0146	1.5267
Uridine	657	168	1.6539	0.0058	1.5263
Palatinose	618	204	2.1142	0.0035	12.6492
Ribonic acid, gamma-lactone	609	117	1.8789	0.0015	1.5008
Conduritol b epoxide	572	217	1.5895	0.0101	1.5621
3-Hydroxy-3-methylglutaric acid	555	247	1.3120	0.0466	1.4388
Methyl heptadecanoate	543	87	1.0250	0.0000	3.9387
4-Hydroxyphenylacetic acid	530	164	1.9489	0.0002	2.3573
Pelargonic acid	509	215	1.9204	0.0040	5.8735
Linoleic acid methyl ester	503	163	1.0131	0.0342	1.9009
Lactose	389	57	1.3997	0.0304	1.3909
5-Aminovaleric acid lactam	384	156	1.2540	0.0020	3.9391
Nornicotine	344	232	1.6329	0.0107	1.7724
Melibiose	333	332	1.0873	0.0471	1.8535
1,3-Diaminopropane	326	292	1.1778	0.0132	4.4758
3-Hydroxynorvaline	309	144	1.7495	0.0047	1.3766
Dihydrotestosterone	278	254	1.1360	0.0309	2.6046
3,5-Dihydroxyphenylglycine	259	260	1.4267	0.0047	3.4809
Saccharopine	211	274	1.1976	0.0058	3.0467
4-Aminophenol	156	240	1.2509	0.0464	3.0828

**Table 2:** Significantly differentially abundant metabolites that were decreased in termites fed a *M. micrantha* leaves diet relative to those fed a pinewood diet.

Metabolites	Similarity	Mass	VIP	*p*-Value	Fold Change
Maltose	818	204	2.2601	3.65 × 10^−5^	9.71 × 10^−7^
Tyrosine	814	218	1.9103	6.80 × 10^−3^	1.37 × 10^−7^
5-Aminovaleric acid	695	174	2.2589	3.68 × 10^−3^	1.65 × 10^−5^
3-Phosphoglycerate	662	227	1.3838	2.65 × 10^−2^	6.05 × 10^−1^
2-Oxoglutarate	651	198	2.2552	2.00 × 10^−3^	1.97 × 10^−5^
Tagatose	646	415	1.8586	7.86 × 10^−4^	4.84 × 10^−1^
6-Phosphogluconic acid	602	318	1.5976	8.79 × 10^−3^	7.27 × 10^−1^
Ergosterol	531	50	1.8937	5.81 × 10^−3^	2.70 × 10^−5^
5-Hydroxy-l-tryptophan	511	290	1.9015	1.29 × 10^−2^	8.17 × 10^−5^
Levoglucosan	490	204	1.5614	3.36 × 10^−5^	1.61 × 10^−1^
dAMP	457	315	2.0136	4.30 × 10^−3^	3.66 × 10^−1^
Progesterone	422	119	1.5917	8.71 × 10^−3^	5.47 × 10^−1^
Thymine	403	270	1.6258	9.82 × 10^−3^	6.81 × 10^−1^
Uridine monophosphate (UMP)	396	211	1.1891	2.90 × 10^−2^	3.67 × 10^−1^
Sphinganine	376	217	1.0614	9.34 × 10^−4^	4.01 × 10^−1^
5-Dihydrocortisol	369	370	1.5367	5.92 × 10^−3^	1.13 × 10^−1^
Glycocyamine	343	274	2.0481	1.22 × 10^−5^	4.90 × 10^−1^
Hydantoin, 5-(4-hydroxybutyl)- 2	304	357	2.0694	5.55 × 10^−5^	4.49 × 10^−1^
l-Kynurenine	304	434	1.0513	2.04 × 10^−2^	5.13 × 10^−1^
Dehydroabietic acid	293	252	1.3628	3.19 × 10^−2^	4.98 × 10^−3^
*N*-Acetyltryptophan	266	290	1.7577	1.63 × 10^−3^	3.39 × 10^−1^
Cholecalciferol	254	213	1.7726	5.76 × 10^−3^	3.83 × 10^−1^
Cyclohexylsulfamic acid	250	303	1.5366	3.44 × 10^−2^	4.79 × 10^−1^
6-Methylmercaptopurine	216	352	1.2724	4.23 × 10^−2^	7.08 × 10^−1^
1-Methyladenosine	212	169	1.6781	7.04 × 10^−3^	5.32 × 10^−1^

**Table 3:** Metabolic pathways identified from the significantly differentially abundant metabolites (SDMs) between termites fed *M. micrantha* leaves (ML) and pinewood (PW) diets.

Pathway	−log10(*p*)	Impact	SDMs ^1^
Glycine, serine and threonine metabolism	4.2426	0.2793	1,3-diaminopropane↑, Pyruvate↑, Serine↑, Glycocyamine↓
Tryptophan metabolism	2.7729	0.2645	5-Hydroxy-l-tryptophan↓, l-Kynurenine↓
Starch and sucrose metabolism	5.8532	0.2516	Glucose-1-phosphate↑, Isomaltose↑, Maltose↓
Galactose metabolism	3.8386	0.2514	Glucose-1-phosphate↑, Lactose↑, Melibiose↑, *myo*-Inositol↑, *N*-Acetyl-d-galactosamine↑, Sorbitol↑, Tagatose↓
Sphingolipid metabolism	3.4365	0.2146	Serine↑, Sphinganine↓
Pyruvate metabolism	3.7194	0.1654	Pyruvate↑
Pyrimidine metabolism	2.8521	0.1417	Uridine↑, Thymine↓, UMP↓
Amino sugar and nucleotide sugar metabolism	1.8373	0.1147	Glucose-1-phosphate↑
Citrate cycle (TCA cycle)	6.5818	0.1049	Pyruvate↑, 2-Oxoglutarate↓
Glycolysis/Gluconeogenesis	3.0713	0.0991	Glucose-1-phosphate↑, Pyruvate↑
Lysine degradation	2.24	0.069	Saccharopine↑, 5-Aminovaleric acid↓
Alanine, aspartate and glutamate metabolism	6.5818	0.0676	Pyruvate↑, 2-Oxoglutarate↓
Fructose and mannose metabolism	1.5513	0.0459	Sorbitol↑
Fatty acid biosynthesis	3.4695	0.0168	Palmitic acid↑
Purine metabolism	2.9125	0.0025	dAMP↓
Tyrosine metabolism	5.2416	0	4-Hydroxyphenylacetic acid↑, Pyruvate↑, Tyrosine↓
Cysteine and methionine metabolism	4.2426	0	Pyruvate↑, Serine↑
d-Glutamine and d-glutamate metabolism	3.9902	0	Glutamic acid↑, 2-Oxoglutarate↓
Butanoate metabolism	3.8176	0	3-Hydroxybutyric acid↑, 2-Oxoglutarate↓
Fatty acid elongation	3.4695	0	Palmitic acid↑
Pentose and glucuronate interconversions	2.2258	0	d-Arabitol↑, Glucose-1-phosphate↑
Pentose phosphate pathway	1.8912	0	Ribose↑

^1^ ↑ and ↓ indicate that the metabolites were upregulated and downregulated in termites fed an ML diet relative to those fed a PW diet, respectively.

## Data Availability

The study did not report any data.

## Supplementary Materials

The following are available online at https://www.mdpi.com/article/10.3390/insects12100927/s1, Table S1: Analysis of the effect of termite colony on bioassays.
