# Supplementary material for: GC/TOF-MS-Based Metabolomics Reveals Altered Metabolic Profiles in Wood-Feeding Termite Coptotermes formosanus Shiraki Digesting the Weed Mikania micrantha Kunth
Source: Insects. 2021 Oct 11;12(10):927. doi: 10.3390/insects12100927 (PMC8537488; doi:10.3390/insects12100927)
Supplement: Supplementary file 1 [file insects-12-00927-s001.zip › insects-1406040-supplementary.pdf]

Table S1. Analysis of the effect of termite colony on bioassays.

(A) Colony effect on termite survival (generalized linear model).

	LR	Chisq	Df	Pr (> Chisq)
Termite survival	19.113	1	1.232e-05	***
Colony	0.100	1	0.7518	

(B) Colony effect on cumulative mortality (generalized linear model).

	LR	Chisq	Df	Pr (> Chisq)
Cumulative mortality	209.664	1	<2e-16	***
Feeding days	117.661	1	<2e-16	***
Colony	0.818	1	0.3648	

(C) Colony effect on diet consumption (one-way ANOVA).

	Sum	Sq	Df	F value	Pr (> F)
Diet consumption	1213.24	1	117.7321	1.807e-06	***
Colony	16.85	1	1.6352	0.233	
Residuals	92.75	9			

Significant codes: '***' represents 0, '**' represents 0.001, '*' represents 0.01, '.' represents 0.05, and blank ' ' represents > 0.1.
